# Global Cancer Burden and Natural Disasters: A Focus on Asia’s Vulnerability, Resilience Building, and Impact on Cancer Care

**DOI:** 10.1200/JGO.19.00037

**Published:** 2019-04-12

**Authors:** Roselle De Guzman, Monica Malik

**Affiliations:** ^1^ St Luke’s Medical Center, Quezon City, Philippines; ^2^ Nizam’s Institute of Medical Sciences, Hyderabad, India

## Abstract

The world has been witnessing more frequent and greater intensity weather-related disasters. Natural disasters hit every continent in the world. Asia has borne the brunt in terms of frequency and the total numbers of people affected. This is mainly because of Asia’s increasing population and its large and varied landmass, with multiple river basins, mountains, flood plains, and active seismic and volcanic zones. The Union for International Cancer Control New Global Cancer Date: GLOBOCAN 2018 has estimated the global cancer burden to have risen to 18.1 million new cases and 9.6 million deaths. Asia constitutes roughly 60% of the world’s population. The region contributes nearly one half of new cancer cases and more than one half of cancer deaths worldwide. This increase in the regional burden of cancer is largely a result of socioeconomic growth and the increasing size and aging of the population. In addition to the increasing cancer cases, the string of natural disasters will cause heavy damage and a great human toll in Asia. Medical care for disaster-affected populations is focused traditionally on the management of immediate trauma and acute infections. For people with noncommunicable diseases, this presents a significant risk. Patients with cancer are especially susceptible to the disruptions that natural disasters can cause. Their special needs are largely neglected. There is a need to refocus and expand disaster risk reduction strategies and resources to include patients with noncommunicable diseases such as cancer, because these conditions are generating the bulk of disability, ill health, and premature death around the globe. Having the world’s biggest burden of cancer, Asia will definitely be facing these challenges.

## INTRODUCTION

With urbanization, climate change, and environmental degradation, the world has been experiencing disasters at a higher intensity and frequency. The recent advances in technology and the accessibility with which news and information travel around the world have made learning about disasters in other countries an almost regular occurrence. The recent typhoons, floods, and earthquakes have led to unimaginable levels of destruction, injuries, and death. The dangers lie in any part of the world. Disasters can occur in places with advanced medical systems and technology such as the United States, Europe, and Japan. However, most of these disasters occur in underserved areas with high population densities where resources and technology are inadequate.

## THE GROWING BURDEN OF CANCER AND THE RISING FREQUENCY AND INTENSITY OF NATURAL DISASTERS IN ASIA

The global cancer burden was estimated to have risen to 18.1 million new cases and 9.6 million deaths in 2018.^1^ Asia constitutes roughly 60% of the world’s population (4.5 billion)^2^ and contributes nearly one half of new cancer cases and more than one half of cancer deaths worldwide. The increase in the regional burden of cancer is largely a result of socioeconomic growth and the increasing size and aging of the population.

Weather-related disasters are becoming increasingly frequent. Over the past 20 years, an overwhelming majority of disasters has been caused by floods, storms, and heat waves. In 2017, 335 natural disasters affected more than 95.6 million people, killing an additional 9,697 and costing a total of $335 billion (US$).^3^ This burden was not shared equally. Asia seemed to be the most vulnerable continent for floods and storms, with 44% of all disaster events, 58% of total deaths, and 70% of total people affected. Over the past decade, China, India, Indonesia, and the Philippines were the countries hit most frequently by natural disasters. The Asian population giants, China and India, were among the hardest hit countries in terms of occurrence, with 25 and 15 events, respectively.^3^ These two nations accounted for more than 3 billion disaster-affected people between 1995 and 2015. This comprises 75% of the global total of 4.1 billion people.^4^

Predictions of more extreme weather in the future almost certainly mean that the world will witness a continued upward trend in weather-related disasters in the years ahead. Patients with cancer constitute a highly vulnerable population when affected by disasters. Medical care for disaster-affected populations is largely based on the management of trauma and acute infections. In such situations, the special needs of people with noncommunicable diseases (NCDs) including cancer are largely neglected. With the increasing number of cancer cases, the string of disasters will cause heavy damage and a great human toll in Asia.

## ASIA’S DEVASTATING NATURAL DISASTERS AND THE VULNERABILITIES OF LOW- AND LOW-MIDDLE INCOME COUNTRIES

The alarming trend shows that natural disasters are now four times more likely to affect people in Asia than in Africa, and 25 times more likely than in Europe. Financially, Asia accounts for almost one half of the estimated global economic cost—close to $1 trillion (US$)—caused by natural disasters since the early 1990s.^5^ This enormous toll is a result of expected fundamental changes in massive population growth in Asia. There are surging populations in low-lying megacities such as Bangkok, Jakarta, and Manila. Millions are forced to move away from historically economically active areas along canals and rivers to more marginal lands along low-lying coastal areas and flood-prone areas in cities. This has left people more vulnerable to storm surges from typhoons and cyclones than those in the higher lands.

## HEAVY BURDEN ON ASIA: MASSIVE HUMAN AND ECONOMIC COST

The world has witnessed devastating natural disasters with extremely high mortality that have occurred in various countries in Asia (Table 1).^6^ These disasters have caused massive damages that have adversely affected human lives and the region’s economy.

**TABLE 1:**
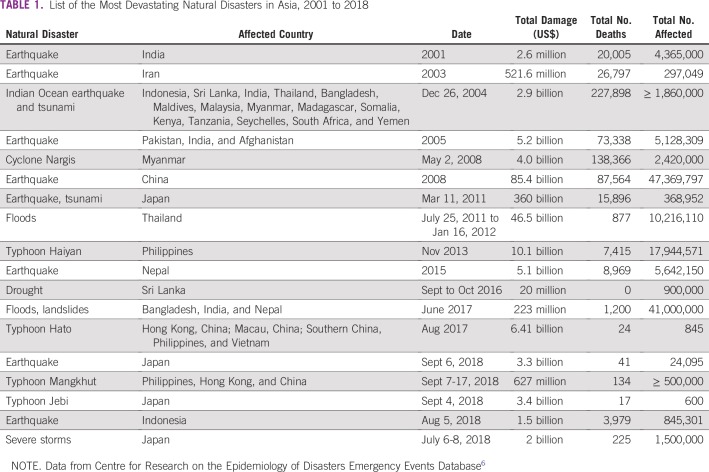
List of the Most Devastating Natural Disasters in Asia, 2001 to 2018

More than a trillion US dollars worth of assets has been lost since 1970. The year with the highest economic losses was 2011, at $400 billion (US$), mainly caused by the earthquake and tsunami in Japan. The United Nations (UN) warns that disasters could cost the Asia-Pacific region $160 billion per year by 2030. Disasters have taken 821,170 lives and have affected approximately 1.4 billion people in Asia since 2000. The impact will rise in the coming decades because of the effects of climate change and lifestyle shifts.

## VULNERABILITIES AND DISASTERS RISKS OF SOUTH ASIA AND LOW- AND LOWER-INCOME ECONOMIES

South Asia is the most exposed region to flooding and is highly exposed to cyclones. Of the world’s total population exposed to floods each year, 64% are in the South Asia region.^7^ The World Disaster Report reveals that this region has the highest affected to euthanized ratio (the correlation between the people affected and the people killed due to natural disasters) in the world.^8^ Asia has a large and varied landmass, with multiple flood plains, river basins, mountains, and active seismic and volcanic zones, placing the region at high risk of natural hazards. High population density, political instability, and weak governance are aggravating the vulnerabilities and exacerbating the hazard impacts in most of these countries.

The World Bank and Columbia University published the Identification of Global Risk Hotspots project in 2005. It used data on historical occurrence, actual disaster events, and zoning of areas to identify those of relatively high risk to lives and livelihoods. One climate change vulnerability index indicates that all seven cities globally classified at extreme risk are in Asia—Dhaka, Manila, Bangkok, Yangon, Jakarta, Ho Chi Minh, and Kolkata.^5^

Disasters affect the livelihoods of those who are already in a susceptible situation, increasing socioeconomic vulnerabilities.

China and the Philippines are among the worst affected by displacement (Table 2).^9^ Some countries still lack the capability to handle disaster-related risks. Some may be capable of forecasting and issuing early warnings; however, the intensity of the disaster can be too devastating to manage. This was witnessed in Japan’s earthquake and tsunami in 2011, Philippines’ typhoon Haiyan in 2013, and Nepal’s Gorkha earthquake in 2015. In most low- and low-middle income countries (LMCs), disaster management is predominantly a reactive response after a disaster, with little or no proactive measures undertaken to improve early warning systems, mitigate risks, and build resilience, even though these may have been incorporated into policy and plan documents. There is also no emphasis on identifying and addressing the issues of people with special needs

**TABLE 2:**
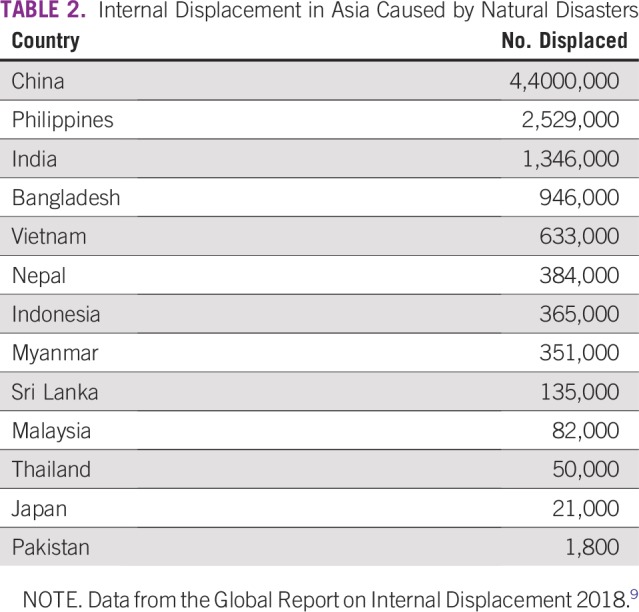
Internal Displacement in Asia Caused by Natural Disasters

## CANCER CARE WHEN DISASTER STRIKES

Although the incidence of global natural disasters has increased steadily over the past several decades, only minor consideration has been given to vulnerable populations, specifically patients with NCDs. Forty million global deaths are a result of NCDs.^10^ It is no longer enough to focus solely on the immediate results of disaster events. The specific needs of vulnerable populations should be strongly addressed when planning, preparing for, and responding to disasters. The WHO Package of Essential Noncommunicable Disease Interventions for primary care in low-resource settings is an innovative and action-oriented response to the challenges noted.^10^ It is a prioritized set of cost-effective interventions that can be delivered to an acceptable quality of care, even in resource-poor settings.

## PATIENTS WITH CANCER ARE VULNERABLE POPULATIONS NEEDING SPECIAL ATTENTION

In a disaster setting, the single most important health need of people with cancer is to ensure uninterrupted access to the needed medication and care. People with NCDs require long-term medical treatment and follow-up. Continuity of care is challenging in an emergency setting. Patients with cancer are especially susceptible to the disruptions that natural disasters can cause. Other factors that may lead to worsening of chronic conditions include degradation of living conditions, compromised immunity, physical injuries, and compromised family or caregiver support. These traumatic events and shocks might exacerbate those with underlying psychological conditions such as depression.

When natural disasters occur, the focus is on survival. For patients with cancer, this is a greater challenge because hospitals, cancer centers, and health care infrastructures may become inaccessible. Medical records and biopsy specimens may be wiped out, submerged underwater, or destroyed. These are critical aspects in oncology care because treatment decision making has now evolved to a more personalized approach. Multidisciplinary team management is an integral part of cancer care, and contact with medical specialists is important in the treatment process. Many cancer drugs require a complex delivery service and set-up and are often administered through a parenteral route over a course of days. Disruptions at any time over the course of cancer care might adversely affect outcomes. Situations may be even more challenging in many LMCs, where cancer care capacity may already be limited before the disaster strikes.

Information on the impact of disaster on cancer care is scarce. There is some evidence that patients are affected negatively by disasters. Available limited studies have shown that interruptions in or prolongation of cancer treatment plans results in poorer locoregional disease control, fewer disease-free survival years, and early death.^11-15^ Advanced planning and preparation can decrease the unnecessary morbidity and mortality for patients with cancer. The importance of the continuity of oncologic care in the wake of a natural disaster cannot be ignored. It is no longer acceptable to overlook this patient population when planning global preparedness and disaster response.

## EMERGENCY PREPAREDNESS PLANS AND THE NEED TO EXPAND RISK REDUCTION AND MITIGATION STRATEGIES FOR PATIENTS WITH CANCER

Acute trauma-related injuries are the leading cause of morbidity and mortality in the immediate aftermath of a disaster. In the weeks after, insufficient nutrition and food supply, poor hygiene and lack of sanitation, and decreased access to health care services further strain any health system. The resilience of the public health infrastructure is an important mitigating strategy. When public health service infrastructure is damaged, access to treatment and care is severely jeopardized, which results in an increased risk of disease exacerbation or even death. Priorities should be set to strengthen the resilience of the public health infrastructure. These include building resilient communication systems and power networks and providing equipment, supplies, transport, sanitation, water, and workforce. To ensure the physical and functional integrity of the public health infrastructure during and after a disaster, attention should be paid to the comprehensive Safe Hospital Framework issued by the WHO.^16^

There should be additional strategies for mitigating the impact of disasters. These include mapping people at risk, maintaining a back-up of medical records, early evacuation of high-risk people, devising mechanisms for referral and transfer to specialized care if necessary, preparing emergency treatment kits, and maximizing sanitation and access to clean water and safe food. There should be storage of back-up medication and equipment in secure locations, availability of power for medical equipment in evacuation centers, and hubs that are set up for oncology treatment and care. Equally important is a mechanism for sharing information among agencies and stakeholders. This supports a workforce strategy that ensures that interagency rapid response systems are in place, there is effective communication across multiple levels, and resources are jointly acquired and pooled to implement the mitigation strategies.

## INTEGRATING CANCER MANAGEMENT INTO PRIMARY CARE DURING NATURAL DISASTERS

Effective management strategies during natural disasters must be able to meet the health needs of affected patients with cancer. The traditional health focus of humanitarian response on immediate trauma and communicable diseases is no longer sufficient to address health needs. By incorporating an oncology component into the immediate disaster response, these vulnerable patients receive the care and compassion they deserve.

Scientific research assessing or addressing the health needs and the short- and long-term impact of disasters on patients with cancer is limited. There is currently no well-recognized and scientifically validated approach to the care of people with cancer in an emergency. There are ways that may help incorporate cancer care during disasters. These may include the use of telemedicine, a close working relationship between public and private sectors, a health professionals’ visit to evacuation centers, and availability of staff trained in disaster management systems.

## PROMOTION OF SELF-MANAGEMENT: NURTURING THE ABILITY OF PATIENTS WITH CANCER TO COPE WITH AND RECOVER FROM DISASTERS

Patient education is an integral part of medical care. In a typical oncology setting, people are educated on prevention, early detection and screening, treatment goals and effects, and rehabilitation and palliative care. Within the same context, patients with cancer must be educated on self-management.

The National Cancer Institute provides seven tips for patients with cancer to prepare them for disasters^17^:

1. Patients should talk with their health care providers about what to do and how they will stay in contact in the event of a disaster;
2. They should make a plan with their family, friends, and neighbors;
3. Each patient should know his or her exact diagnosis, cancer stage, medications, and treatment cycle;
4. If on a clinical trial, he or she should know its details;
5. Each patient should make sure to have his or her health care provider’s contact information available, even in emergencies;
6. If patients have insurance, they should make sure to carry their insurance cards; and
7. Each patient should make a kit with the items that he or she may need.

Although the tips are written for patients with cancer, they are comprehensive and are generally in line with the recommendations for patients with other chronic conditions.^18^ In resource-limited nations, however, poverty, illiteracy, and poor access to health care are significant barriers toward implementing these measures.

**FIG 1:**
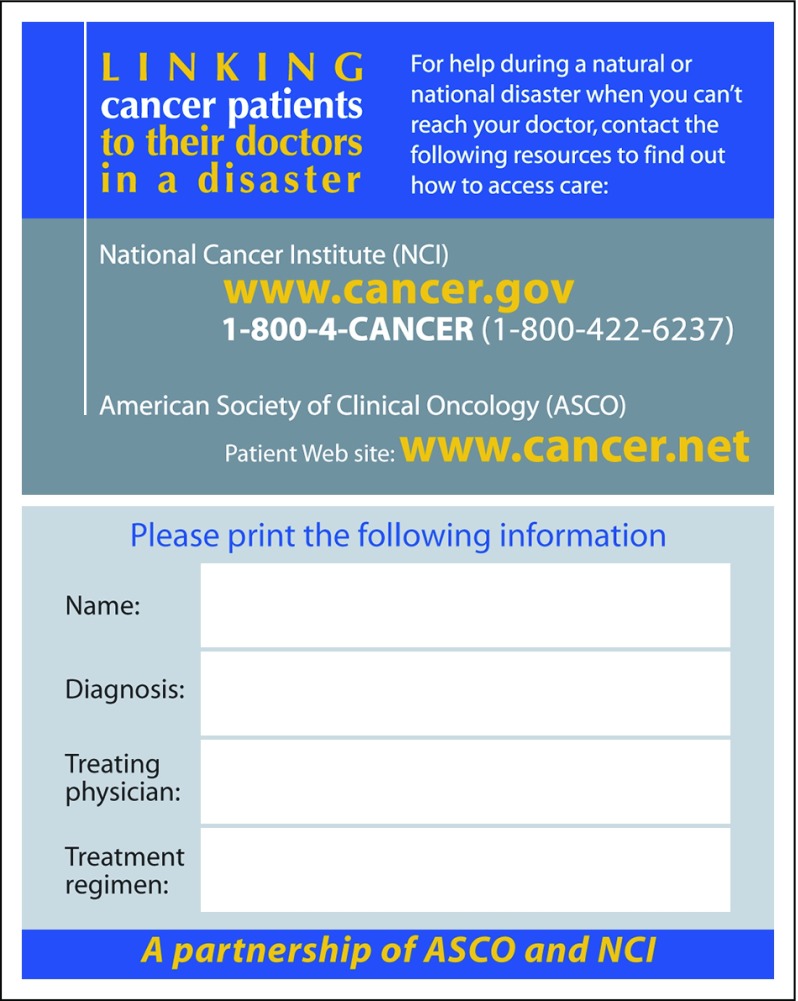
ASCO and National Cancer Institute patient information wallet card.

Patients need to know their history and have pertinent information stored, both on paper and electronically. Medical records must be made disaster proof. Electronic medical records that are accessible among facilities can make the health care process easier. An emergency contact person should be identified and entered in a cellular phone. A wallet card should be kept, summarizing the complete diagnosis and disease stage, treatment details, and oncologist information. The National Cancer Institute, in partnership with ASCO, recommends that patients carry a wallet card (Fig 1)^19^ with basic information about their cancer treatment and emergency contact information, to access care in case patients cannot reach their physician in an emergency or during a natural disaster.^17^

Patients with cancer have more than enough to think about. They need to focus on being able to continue treatment and the healing process. They should use checklists for self-monitoring of physical conditions to detect any deterioration during or after a disaster (eg, dizziness or palpitations may mean anemia; fever may mean febrile neutropenia or active infection). The checklist should also include advice on nutrition and prevention of infections in a disaster setting.

Strong preparedness on the patient’s part must be complemented and supported by a resilient public health care system. A registry and health surveillance system should be established to record essential information on the health needs of people with cancer. The information should include the size of the targeted population, address or location, health conditions, capacities, and medication and treatment needs. The system should also record morbidity and mortality information in the post-disaster stage. This information can assist in determining prevalence rates, disease patterns, and processes to build emergency preparedness. These can also be used in conducting needs assessment, reaching out to these vulnerable people during disasters, monitoring post-disaster health outcomes and long-term health effects, and effectiveness of the post-disaster response.

## A COMPREHENSIVE GLOBAL DEVELOPMENT FRAMEWORK: FROM PREVENTION TO DISASTER RISK REDUCTION, RESILIENCE BUILDING AND SUSTAINABLE DEVELOPMENT

In 1987, the World Commission on Environment and Development highlighted the devastating impacts of natural disasters on economic and social development. It pointed out the need for national resilience building.^20^ The UN General Assembly adopted an International Framework of Action, which called on governments, especially those of developing countries, to integrate economic and insurance policies for disaster prevention and mitigation programs into national development agenda.^20^

Different UN agencies and national governments proposed separate but interrelated agreements that compose the comprehensive global development frameworks. These include the 2030 Agenda for Sustainable Development, the Sendai Framework for Disaster Risk Reduction 2015-2030, the Paris Agreement under the UN Framework Convention on Climate Change, the Agenda for Humanity, the New Urban Agenda, and the Addis Ababa Action Agenda of the Third International Conference on Financing for Development.^20^ Sustainable development is the core of the global development frameworks.

Natural disasters displace people and increase their socioeconomic vulnerabilities. Globally, more than 60 million people were displaced by disasters between 2013 and 2015.^21^ A great majority were in the Philippines, China, India, Nepal, Bangladesh, Pakistan, and Myanmar. The aim of the global agenda for sustainable development is for these countries to further their resilience.^20^

## ACTION FOR REGIONAL COOPERATION: THE ECONOMIC AND SOCIAL COMMISSION FOR ASIA AND THE PACIFIC AND A FRAMEWORK FOR RESILIENCE OF ASSOCIATION OF SOUTHEAST ASIAN NATIONS COUNTRIES

The Economic and Social Commission for Asia and the Pacific (ESCAP) is the UN’s largest regional development arm and intergovernmental platform. It has 53 member states and nine associate members.^21^ ESCAP works to overcome some of the region’s challenges by providing capacity-building, results-oriented projects and technical assistance to member states in various areas that include social development, information and communications technology, and disaster risk reduction. ESCAP promotes cooperation among member countries to achieve sustainable development. Its focus is managing globalization through programs in environmentally sustainable development, trade, and human rights.

The Association of Southeast Asian Nations adopted a Declaration on Institutionalizing the Resilience of its organization and its Communities and Peoples to Disasters and Climate Change.^21^ This declaration highlighted the importance of integrating disaster risk management and climate change adaptation into policies, strategies, and projects.^20^

## ACTION POINTS FOR BUILDING RESILIENCE AT A NATIONAL LEVEL

Some countries in Asia had initiated steps toward resilience building before adopting sustainable development goals. Japan prepares an annual parliament report on the status of disasters and budgetary allocations for disaster risk reduction programs. The Indian Government has formulated a National Disaster Management Plan aligned with the Sendai Framework. The Philippines has integrated climate change adaptation and disaster risk reduction into its development policies, plans, and budgets. China has a comprehensive Atlas of Natural Disasters that is used for developing disaster prevention and reduction plans.^22^

The least developed countries in Asia have prepared National Adaptation Programs of Action. Most countries have prepared National Capacity Self-Assessments and National Communications. Many have also started their National Adaptation Plans. Sri Lanka was the region’s first country to submit a plan to the UN’s Framework Convention on Climate Change.^23^

## ASIA’S FUTURE DISASTER RISKS AND THE CHALLENGES AHEAD

Natural disasters do not discriminate. It is impossible to predict where and when the next disaster will take place. Between 2015 and 2030, it is estimated that the population in Asia’s extreme-risk areas will grow by 35% to 50% in various cities.^22^

Forty percent of global economic losses from disasters will occur in Asia in the decade spanning 2020 to 2030, with the region’s largest economies—Japan, South Korea, China, and India—being the worst affected. Poorer nations like Bangladesh, Cambodia, Myanmar, the Philippines, and Laos, however, will have less capacity to either prepare for disasters or respond to their aftermath, thus putting far more people at risk of death or displacement.

A more comprehensive approach to cancer management in emergencies is an important but neglected aspect of humanitarian response. Oncology care needs to be integrated into standard operating procedures. Governments and communities in Asia need to recognize the increasingly endemic nature of disasters, the burden of cancer, and how these can affect health systems and health and derail economic growth and development. Investment in increasing resilience to natural disasters and global cancer control will be far reaching if these are well integrated into development policies, strategies, and assistance programs.

## ASIA’S CONSIDERABLE POTENTIAL TO HARNESS OPPORTUNITIES AND OVERCOME THE CHALLENGES

Asia has significant potential to build a resilient and sustainable future to achieve the goals outlined in global development frameworks. Despite its constraints and difficulties, the Asia Pacific economy is continually growing. The economic growth of China and India is relevant to achieving millennium development goals.

Asia has a strong foundation in disaster risk management. Countries have developed national frameworks on disaster risk reduction and have strengthened systems for disaster preparedness and response. Various stakeholders have been active throughout the region, pursuing the agenda of resilience through networks and federations.

## THE ROLE OF MEDICAL SOCIETIES AND ONCOLOGY PROFESSIONALS

Scientific societies could play an extremely important role in the different phases of disaster management, from prevention, mitigation, and preparedness, to response and recovery. The challenge of NCDs has been recognized in the Sendai Framework, which is complementary to and builds on the WHO Global Action Plan for the Prevention and Control of NCDs–2013-2020.^24^ Medical societies can collaborate on risk reduction and prevention strategies in support of the WHO in its efforts to improve preparedness for disasters. They can forge and maintain strong relationships with relevant organizations (ie, government agencies, ministries of health, and nongovernment groups). This can ensure active involvement in interagency planning efforts that include designing appropriate coordination protocols, developing an integrated communication plan, and oncology care.

Health professionals can take a lead role in coordinating with other health providers such as the Red Cross, emergency physicians and staff, and others engaged in planning for and responding to emergencies. They can foster a better training of health care providers in responding to the special needs of people with cancer. Oncology professionals can participate in training and educational programs to stay abreast of evolving disaster management systems so they may help educate the community through advocacy programs on prevention of infectious diseases and control efforts that may mitigate complications and life-threatening conditions during disasters. Oncology societies can also help ensure that other stakeholders have the appropriate programs, supplies, and equipment in place. Partnerships can support innovative technology such as telemedicine as a way of providing uninterrupted team-based care that is a critical aspect of oncology care.

Collaborative efforts between oncology societies and state or local public health groups can ensure the supply of a dedicated and specialized workforce. The creation of a network or taskforce including oncologists, nurses, therapists, and psychologists would be able to support not only affected people, but also affected colleagues and other health care staff who may be in the affected areas and may themselves experience extreme stress and burnout in responding to disasters.

Delivery of quality oncology care is always a challenge in LMCs, and more so in a restricted situation during a disaster event. Oncology societies can further assist in developing protocols to ensure appropriate resource allocation. Application of cancer-specific resource-stratified guidelines would help optimize cancer care in settings of limited resources. This may ensure that services are provided equitably and impartially, consistent with ethical and legal standards relevant in the disaster event and based on the vulnerability and needs of the individuals and communities affected by disaster.

Finally, because of the limited information on the impact and health effects of natural disasters, support for research in these areas should be pursued. Disaster plans usually do not include provisions for data collection. Inadequate documentation of the health impacts greatly hinders health planning and measurement outcomes. This may be a collaborative effort among medical societies and other health sectors at the regional, national, or local level.

The global cancer burden and a string of natural disasters are challenges Asia is currently facing. These are expected to cause additional heavy damage and a great human toll in this region in the near future. Asia’s vulnerabilities and disaster risks are clear. Resilience building, together with global development frameworks, regional cooperation, and national action points, are strengthening the mitigating measures. Patients with cancer are a vulnerable population. The significance of integrating cancer care into disaster planning must be recognized. A comprehensive approach to cancer management during emergencies is an important but neglected aspect of the humanitarian response. Oncology care needs to be integrated into different phases of disaster management, from prevention, mitigation, and preparedness, to response and recovery.

## Data Availability

The following represents disclosure information provided by authors of this manuscript. All relationships are considered compensated. Relationships are self-held unless noted. I = Immediate Family Member, Inst = My Institution. Relationships may not relate to the subject matter of this manuscript. For more information about ASCO's conflict of interest policy, please refer to www.asco.org/rwc or ascopubs.org/jgo/site/misc/authors.html. **Honoraria:** Roche Oncology (Philippines), AstraZeneca, Novartis, Merck Serono, MSD, Boehringer-Ingelheim **Consulting or Advisory Role:** Roche, Bayer AG, Novartis, Merck Serono **Travel, Accommodations, Expenses:** Boehringer-Ingelheim, Hospira (Philippines), Roche (Philippines), Merck Sharp & Dohme, Eisai No other potential conflicts of interest were reported.
